# Experimental Research on Manson–Coffin Curves for the Frame Material of an Unconventional Vehicle

**DOI:** 10.3390/ma15051768

**Published:** 2022-02-26

**Authors:** Miroslav Blatnický, Ján Dižo, Milan Sága, Marek Brůna, Milan Vaško

**Affiliations:** 1 Department of Transport and Handling Machines, Faculty of Mechanical Engineering, University of Žilina, Univerzitná 8215/1, 010 26 Zilina, Slovakia; miroslav.blatnicky@fstroj.uniza.sk (M.B.); jan.dizo@fstroj.uniza.sk (J.D.); 2 Department of Applied Mechanics, Faculty of Mechanical Engineering, University of Žilina, Univerzitná 8215/1, 010 26 Zilina, Slovakia; milan.saga@fstroj.uniza.sk (M.S.); milan.vasko@fstroj.uniza.sk (M.V.); 3 Department of Technological Engineering, Faculty of Mechanical Engineering, University of Žilina, Univerzitná 8215/1, 010 26 Zilina, Slovakia

**Keywords:** unconventional steering mechanism, tricycle, frame, material fatigue, optimization

## Abstract

The submitted research paper describes the fundamental findings in terms of multiaxial fatigue of the basic material EN AW6063 and its welds for implementation in the frame design of an unconventional vehicle. It also includes a briefly-presented conceptual design of a technical solution for optimizing the functionality of a steering mechanism in a patented unconventional vehicle, designed by the authors to increase the cornering stability of a vehicle–tricycle. The most important part of this article is the description of the ongoing research and the results of multiaxial fatigue (bending–torsion combination) of the structural material for the construction of the vehicle frame. The research in this area is important due to the increased load on the frame during operation caused by the unconventional steering mechanism. The measured and constructed Manson–Coffin curves indicate that the use of material EN AW6063 is possible for this vehicle in terms of multiaxial stress. This also applies to the material affected by the technology in the frame production (TIG welding). A higher fatigue of the basic material was observed at a 90° phase shift. The difference between the 0° and 90° phases practically makes up approximately 10 to 15% of the difference in the cycle numbers with the same deformation amplitude. At the same time, the measured results show that the phase shift between loads will not play such an important role in welded joints of aluminum alloy EN AW6063. When comparing the dependences with a constant deformation amplitude in bending and in torsion, it can be said that the bending stress will react more to even a small change in the deformation amplitude. Bending has been proven to be the more dominant component of the total deformation amplitude in multiaxial tests. In terms of low-cycle multiaxial fatigue (up to 5 × 105 cycles), a higher fatigue of the basic material is found in comparison with the weld. At lower deformation amplitudes, a higher fatigue of the welded material is detected.

## 1. Introduction to Problem Statement

The presented vehicle, a so-called “E3-cycle”, is a three-wheeled vehicle with symmetrically arranged wheels. It has a single (steered) front wheel and two rear wheels. This type of arrangement in conventional vehicles is called Delta or by abbreviation 1F2R (1 front, 2 rear). The main advantage of this type is better performance utilization. The drive element transmits the torque to the rear wheelset, which is then distributed to both wheels. This is a rear-wheel drive that has good traction properties, regardless of the conditions. It can be utilized in a vehicle designed for fast driving on a straight track, e.g., for sports purposes. Another advantage of the 1F2R is the ability to use a simple steering mechanism (similar to a bicycle or motorcycle), thereby reducing manufacturing costs. When using a steering mechanism designed for a two-track vehicle on a three-wheeled vehicle, there is a problem with the distribution of inertial forces. In practice, this means that before cornering, it is necessary to significantly reduce the driving speed and prevent positive (increasing speed) or negative (braking) acceleration during cornering. This is done to prevent the vehicle from tilting or tipping over. This fact significantly reduces the overall safety of the vehicle [1,2].

The stability problem can be solved by tilting the vehicle frame. Along with the frame, it is possible to tilt any number of wheels. This helps to distribute the centrifugal forces that arise when driving through curves. The design solution of a tilting tricycle is complex and thus increases manufacturing costs. Nevertheless, tilting the vehicle brings greater safety, which is an essential aspect in the quality assessment of the vehicle [3,4,5,6].

**Figure 1:**
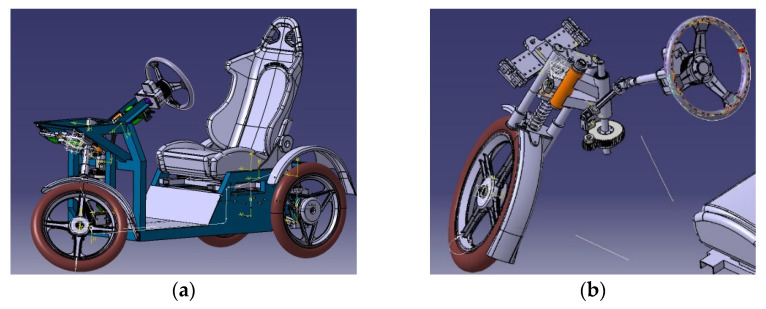
(**a**) A 3D CAD model of the unconventional three-wheeled vehicle E3-cycle; (**b**) An assembly of the original patented steering mechanism.

There are a large variety of design solutions for tilting tricycles [7,8,9,10]. The authors introduced the design for the unconventional steering mechanism of the E3-cycle in a pre ious paper [11]. The designed frame consists of aluminum profiles made of the material EN AW6063 (Figure 1a).

The special suspension of the E3-cycle front wheel enables a wheel twisting, its rotation as well as inclination. This means that the stated design gets the vehicle in a more advantageous position in terms of stability. Thanks to the obtained wheels twisting, the vehicle better overcomes the lateral load caused by inertial forces. However, in the inclined state, there is increased pressure in the arch and chassis of the vehicle [11], which is transmitted through the frame to the wheels. The resulting load thus causes a change in the perpendicular compressive force acting on the contact patch between the tire and the roadway. For these reasons, it was necessary to focus research on determining the fatigue of the vehicle frame. Approximately 80–90% of all structural failures arise due to fatigue [12,13,14]. The issue of uniaxial fatigue and frame design was addressed also in [11,15], where the vehicle frame lifetime was examined using uniaxial fatigue tests. In this case, however, the research deals with a combination of these loads. The overall optimization of the designed vehicle is thus carried out not only in terms of functionality (removal of the singularity of the patented mechanism in the straight direction), but also the lifespan of the frame design (multiaxial fatigue of the frame). Vehicle (as well as E3-cycle) frames are repeatedly in a state of stochastic excitation while driving [16,17]. As a result, repetitive stresses form in the frames and these can over time cause microscopic damage to the material. With continued loading, this leads to a fracture of the part [18]. The fact that the frame of the E3-cycle is a very complex design and that welding technology was used for its manufacture also contributes to this process. Therefore, it was necessary to work on the findings of how the welding of the basic material (EN AW6063) affects fatigue [19].

The authors investigated the effects of welding on the basic material in [11]. Other research also explains how the welding process can affect static strength, residual stresses, and deformation after welding [20,21,22,23].

The manufacture of a real and safe prototype of the E3-cycle requires the input fatigue characteristics of the frame material. These characteristics were obtained empirically using multiaxial fatigue tests. The experiments were conducted on a unique device, which the authors presented in [15,24,25,26].

The vehicle (Figure 1a) consists of several structures such as the frame, the rear axle, the drivetrain mechanism consisting of an electric motor [27,28], the steering mechanism and the seat. The rear axle and the drivetrain consist of an electric motor, a differential gear, coupling shafts, a wheel hub, wheels, axle arms, drum brakes and an adjustable shock absorber. 

With regard to development and innovation, the most important part is the steering mechanism (Figure 1b) [29]. The steering wheel is connected to the frame by the steering wheel holder, in which a tie rod is fixed. The tie rod transmits the force through the cross joints to the pinion, which transmits the torque to a larger toothed wheel using a toothed gear. This creates a strengthening effect and reduces the force required to induce a change of direction.

It should be added that in other research prepared by the authors, which deals with the dynamic analysis of this vehicle through the Simpack program (Dessault Systèmes, Velizy-Villacoublay, France), the results did not show any presence of nonlinearities or singularities of the designed mechanism. This was observed after the construction of a real prototype and its driving tests. It was found that when driving in straight direction, the steered front wheel oscillates around the center position. This condition did not completely make it impossible to drive the vehicle, but its implementation into mass production would not be possible due to the lack of stability and safety. The ride comfort is also reduced. The compound motion of the wheel (rotation, shift and inclination [11]) causes a difference in the position of the vehicle’s center of gravity in the vertical direction. This means that the most energetically favorable position of the mechanism is in its extreme positions. The extreme positions of the mechanism occur when driving through a left or right curve. Returning to the straight direction is therefore more difficult than the movement which caused the direction of drive. This is unacceptable with regards to the standard. Due to this, the optimization of the steering mechanism was investigated. The only requirement for the design was for the mechanism to have an advantage in production cost compared to other unconventional solutions.

**Figure 2:**
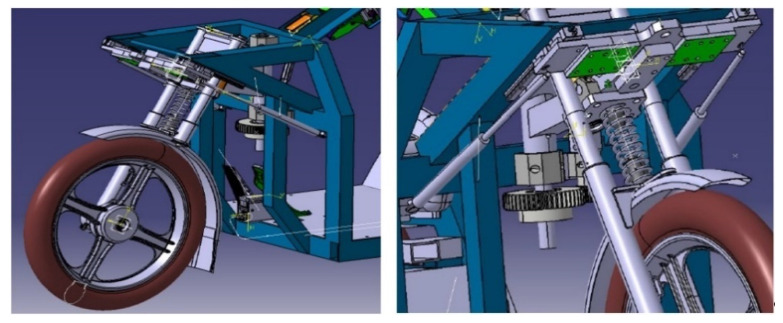
Different views of the 3D CAD models of the steering mechanism optimization by adding telescopic struts.

The optimization of the design consisted in adding telescopic struts to the original design (Figure 2). A similar system of stabilizing the front wheel is used in practice for sports modifications of road motorcycles [30,31,32,33,34]. The choice of struts for this design is limited by the force expended by the driver. However, this force must be sufficient to ensure reciprocating motion of the front wheel. The force exerted by the driver can be changed by means of the gear ratio between the pinion and the toothed wheel. The struts are attached to the frame at one end with a fixed joint. On the opposite side, they are attached to the linear bearing guide. The effect of the struts is manifested by a compressive force that helps the steering fork to return to the straight direction.

**Figure 3:**
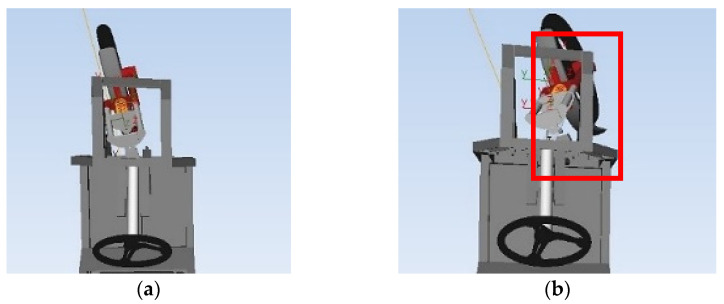
A documentation image for a comparison of the way curves are followed by a three-wheeled vehicle: (**a**) With a standard steering system (only rotation); (**b**) With a newly designed steering system (rotation, shift, pitch).

The advantage of this design is the simplicity of the execution. The design does not require complex technical interventions in the original design. The disadvantage of this model is the resistance when inducing a change of direction, which will be automatically compensated by the law of conservation of energy. Figure 3 shows a comparison of the basic difference in terms of the way of driving through the curve by a three-wheeled vehicle with the standard steering system (Figure 3a) and newly designed steering system with improved front wheel mobility (highlighted by red square in Figure 3b), models were created using the Simpack program.

The advantages of the new type of wheel steering in comparison with the conventional method lie in the difference of the front fork suspension design. The newly designed suspension system causes the fork to deflect to the outer side of the curve being followed [11]. The result of this process will be an increase in the overturning stability of the E3-cycle.

To illustrate, if the vehicle is following a left curve, the front wheel will move to the right from its center position during the driving maneuver. The wheel therefore always moves in the direction of the centrifugal force. This increases the stabilizing moment effect from the vehicle’s gravity by increasing the distance to the tipping axis (line joining front and rear wheel).

## 2. Materials and Methods

**Figure 4:**
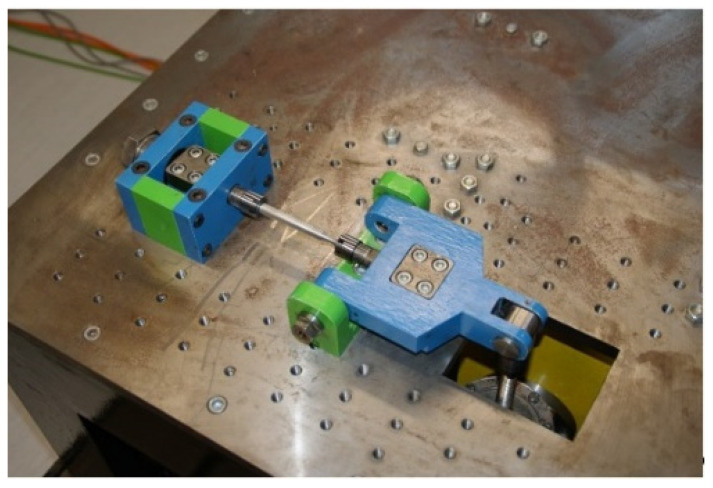
Multiaxial fatigue test equipment.

The experimental part of the paper deals with multiaxial fatigue tests of the frame material of the vehicle in question. The principle of operation of the testing device, the so-called “Fatigue testing system” (Figure 4), was elaborated in detail in articles [15,24]. In article [11], the authors also presented the mechanical properties of the structural material EN AW6063. 

The initial design of the sample shape resulted from the geometry of the testing device and the requirement for simplicity in sample symmetry. The inspiration for deriving the shape of the sample was the measurement of high-cycle fatigue of materials, for example in [35,36].

**Figure 5:**
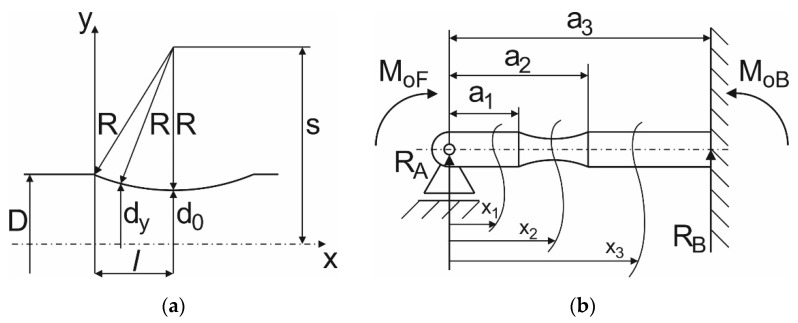
(**a**) Change of the cross-section of the specimen depending on the length of the sample; (**b**) A simplified diagram of the loading mechanism of the testing conditions.

In another design, mathematical modeling was used to maximize the effect of the notch (Figure 5a) as a stress concentrator. The task was to determine the distribution of the maximum stress along the length of the sample. From Figure 5b it is apparent that the calculation of stresses will be relatively simple (constant cross-section) in parts *a*_1_ and *a*_3_ of the sample. In section *a*_2_ the cross-section changes, function s is constant and is determined by formula (1). The boundary conditions of the test specimen defined the static indeterminacy—for the overall solution it is necessary to find the value of the statically indeterminate quantity, e.g., *R_A_*:

(1) $$s=\frac{d_{o}}{2}+R,$$

From the geometry in Figure 5a for length *l* (2):

(2) $$l=\sqrt{R^{2}-{\left(s-\frac{D}{2}\right)}^{2}},$$

and for change of the cross-section *d_y_* (3):

(3) $$d_{y}=s-\sqrt{R^{2}-{\left(a_{1}+l-x_{2}\right)}^{2}},$$

The moment of inertia of the constant circular cross-section of the sample is determined by the known formula (4):

(4) $$J_{z}\left(x_{1}\right)=J_{z}\left(x_{3}\right)=\frac{\pi \cdot D^{4}}{64},$$

and the changing cross-section of the sample (the neck) is determined by the derived formula (5):

(5) $$J_{z}\left(x_{2}\right)=\frac{\pi \cdot d_{y}^{4}}{64}=\frac{\pi \cdot r_{y}^{4}}{4}=\frac{\pi \cdot {\left[s-\sqrt{R^{2}-{\left(a_{1}+l-x_{2}\right)}^{2}}\right]}^{4}}{4}.$$

The boundary conditions of the calculation are determined by formulas (6)–(8):

(6) $$\begin{array}{c} 0\leq x_{1}\leq a_{1}\\ M_{o}\left(x_{1}\right)=M_{oF}+R_{A}\cdot x_{1} \end{array}$$

(7) $$\begin{array}{c} 0\leq x_{2}\leq a_{2}\\ M_{o}\left(x_{2}\right)=M_{oF}+R_{A}\cdot x_{2} \end{array}$$

(8) $$\begin{array}{c} 0\leq x_{3}\leq a_{3}\\ M_{o}\left(x_{3}\right)=M_{oF}+R_{A}\cdot x_{3} \end{array}$$

From Castigliano’s theorem (9):

(9) $$\begin{array}{l} U=\int_{\left(l\right)}\frac{M_{o}^{2}\cdot dx}{2\cdot E\cdot J_{z}}\\ 0=\int_{0}^{a_{1}}\frac{M\left(x_{1}\right)}{E\cdot J_{z}\left(x_{1}\right)}\cdot \frac{\partial M\left(x_{1}\right)}{\partial R_{A}}\cdot dx_{1}+\int_{a_{1}}^{a_{2}}\frac{M\left(x_{2}\right)}{E\cdot J_{z}\left(x_{2}\right)}\cdot \frac{\partial M\left(x_{2}\right)}{\partial R_{A}}\cdot dx_{2}+\int_{a_{2}}^{a_{3}}\frac{M\left(x_{3}\right)}{E\cdot J_{z}\left(x_{3}\right)}\cdot \frac{\partial M\left(x_{3}\right)}{\partial R_{A}}\cdot dx_{3}\\ 0=\int_{0}^{a_{1}}\frac{M_{oF}+R_{A}\cdot x_{1}}{E\cdot J_{z}\left(x_{1}\right)}\cdot x_{1}\cdot dx_{1}+\int_{a_{1}}^{a_{2}}\frac{M_{oF}+R_{A}\cdot x_{2}}{E\cdot J_{z}\left(x_{2}\right)}\cdot x_{2}\cdot dx_{2}+\int_{a_{2}}^{a_{3}}\frac{M_{oF}+R_{A}\cdot x_{3}}{E\cdot J_{z}\left(x_{3}\right)}\cdot x_{3}\cdot dx_{3}\\ 0=\int_{0}^{a_{1}}\frac{M_{oF}\cdot x_{1}}{E\cdot J_{z}\left(x_{1}\right)}\cdot dx_{1}+\int_{a_{1}}^{a_{2}}\frac{M_{oF}\cdot x_{2}}{E\cdot J_{z}\left(x_{2}\right)}\cdot dx_{2}+\int_{a_{2}}^{a_{3}}\frac{M_{oF}\cdot x_{3}}{E\cdot J_{z}\left(x_{3}\right)}\cdot dx_{3}+\\ +\int_{0}^{a_{1}}\frac{R_{A}\cdot x_{1}^{2}}{E\cdot J_{z}\left(x_{1}\right)}\cdot dx_{1}+\int_{a_{1}}^{a_{2}}\frac{R_{A}\cdot x_{2}^{2}}{E\cdot J_{z}\left(x_{2}\right)}\cdot dx_{2}+\int_{a_{2}}^{a_{3}}\frac{R_{A}\cdot x_{3}^{2}}{E\cdot J_{z}\left(x_{3}\right)}\cdot dx_{3} \end{array}$$

For a statically indeterminate quantity *R_A_* from Equation (9) we then get:

(10) $$R_{A}=\frac{\int_{0}^{a_{1}}\frac{64\cdot M_{oF}\cdot x_{1}}{E\cdot \pi \cdot D^{4}}\cdot dx_{1}+\int_{a_{1}}^{a_{2}}\frac{4\cdot M_{oF}\cdot x_{2}}{E\cdot \pi \cdot {\left[s-\sqrt{R^{2}-{\left(a_{1}+l-x_{2}\right)}^{2}}\right]}^{4}}\cdot dx_{2}+\int_{a_{2}}^{a_{3}}\frac{64\cdot M_{oF}\cdot x_{3}}{E\cdot \pi \cdot D^{4}}\cdot dx_{3}}{\int_{0}^{a_{1}}\frac{64\cdot x_{1}^{2}}{E\cdot \pi \cdot D^{4}}\cdot dx_{1}+\int_{a_{1}}^{a_{2}}\frac{4\cdot x_{2}^{2}}{E\cdot \pi \cdot {\left[s-\sqrt{R^{2}-{\left(a_{1}+l-x_{2}\right)}^{2}}\right]}^{4}}\cdot dx_{2}+\int_{a_{2}}^{a_{3}}\frac{64\cdot x_{3}^{2}}{E\cdot \pi \cdot D^{4}}\cdot dx_{3}}.$$

The bending stress in the individual cross-sections will then be (11):

(11) $$\sigma \left(x_{i}\right)=\frac{M_{o}\left(x_{i}\right)}{W_{o,i}};$$

where *W_o_* is the section modulus in bending and its general form of equation for a circular cross-section is as following (12):

(12) $$W_{o,i}=\frac{\pi \cdot d_{i}^{3}}{32}=0.1\cdot d_{i}^{3}.$$

**Figure 6:**
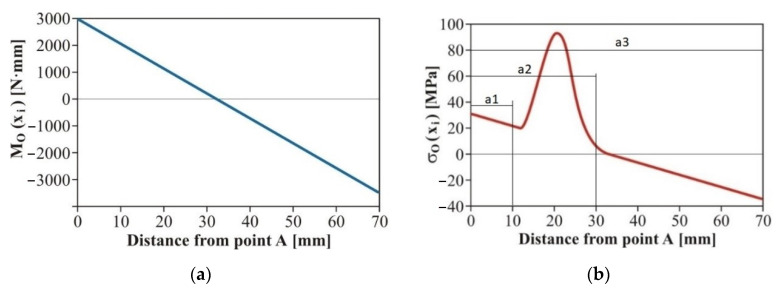
(**a**) Dependence of the bending moment induced by the Fatigue testing system along the length of the specimen; (**b**) Distribution of the normal stress along the length of the specimen loaded by the Fatigue testing system.

The dependence of the bending moment of the sample induced by the testing device is shown in Figure 6a. The bending moment values served as an input for the quantification of bending stresses *σ* calculated by Equation (11) (Figure 6b). In principle, normal stresses (*σ*) are stresses in the main stress direction. The results of Equations (1)–(12) show that it is necessary to change the geometry of the symmetrically designed test specimen.

**Figure 7:**
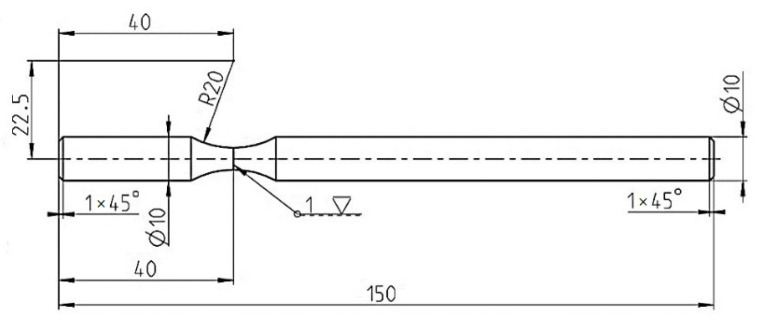
Geometry of the test specimen used to determine the multiaxial fatigue of the frame material of the unconventional vehicle on the designed testing device.

Figure 6a shows that when a bending load is applied, the maximum bending moment is located in close proximity to the specimen grip holder (distance values 0 and 70 mm—the value is fixed and results from the geometry of the testing device. The value 70 mm is therefore the perpendicular distance between the collets). The maximum effect of the notch during fatigue tests can be achieved by moving it from the center of the specimen (an original symmetrical design) to the areas located closer to the bending grip. The calculation indicated the need to move the notch up to the grip, which bends the specimen. Then the initiation and extension of cracks occurs at the monitored area. Respecting the geometry of the testing device, the mathematical model and the simplicity of the experimental specimens resulted in the optimal shape design of the test specimens as shown in Figure 7.

In addition to the testing device, the papers [1,4] also dealt with the mechanical production of specimens and the influence of welding on its properties. This paper presents experimental determinations of fatigue characteristics of aluminum alloy EN AW 6063. Due to the nature of the testing device, the loading was realized in controlled deformation amplitude (Manson–Coffin). The testing conditions allow a large number of combinations (a total of 18 levels [15]) of deformation amplitudes of both stresses (bending, torsion) along with combinations of phase shift. For the measurement methodology, it was determined that the phase shift will be measured at two states, namely at a phase shift of 0° and 90°. The number of 18 positions for each of both deformation amplitudes is graduated and thus reduced to 6 levels, for which will be created individual combinations.

It is also important to verify the results. This is carried out using the Fatigue Calculator—a program from the eFatigue website [37]. To estimate the fatigue under combined stress, it is necessary to set the system to multiaxial low-cycle fatigue. For the relevance of the comparison of experimental and calculated results, the same conditions of the combined loading method of the test specimen—sinusoidal cyclic stress—were chosen. Next, the values of deformations in the required units are entered into the Fatigue Calculator from the calculated tensor, namely for *σ_xx_* and *τ_xy_*. As with experimental measurements, a symmetrical frequency of 30 Hz load cycles with a phase shift of 0° and 90° is used. The Fatigue Calculator displays the results for different damage models. In this case (low-cycle fatigue), these were the cycle numbers to the fracture for the Fatemi–Socie, SWT, Brown–Miller and Liu models.

**Figure 8:**
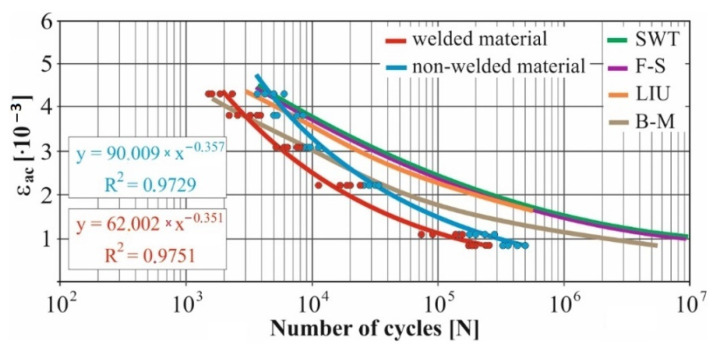
Comparison of *ε_ac_*–*N_f_* curves of selected fatigue criteria and experimentally obtained values, results of low-cycle multiaxial fatigue, constant deformation in torsion *γ_ac_* = 2.5 × 10^−3^, phase shift of loads *φ* = 0°.

**Figure 9:**
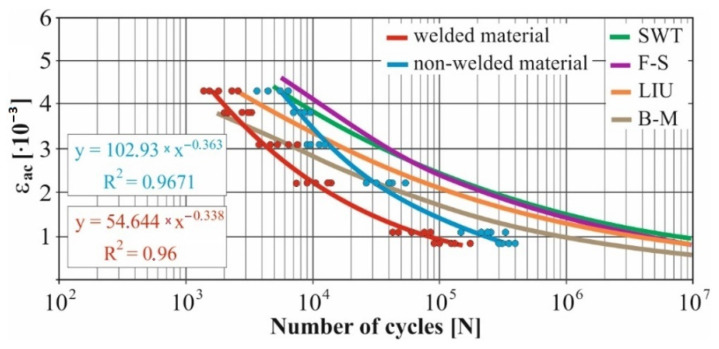
Comparison of *ε_ac_*–*N_f_* curves of selected fatigue criteria and experimentally obtained values, results of low-cycle multiaxial fatigue, constant deformation in torsion *γ_ac_* = 2.5 × 10^−3^, phase shift of loads *φ* = 90°.

**Figure 10:**
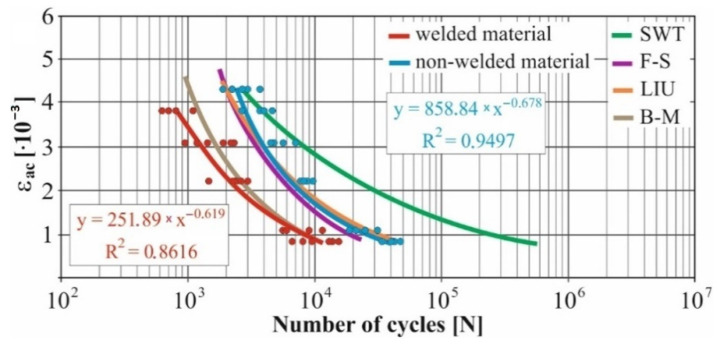
Comparison of *ε_ac_*–*N_f_* curves of selected fatigue criteria and experimentally obtained values, results of low-cycle multiaxial fatigue, constant deformation in torsion *γ_ac_* = 5 × 10^−3^, phase shift of loads *φ* = 0°.

**Figure 11:**
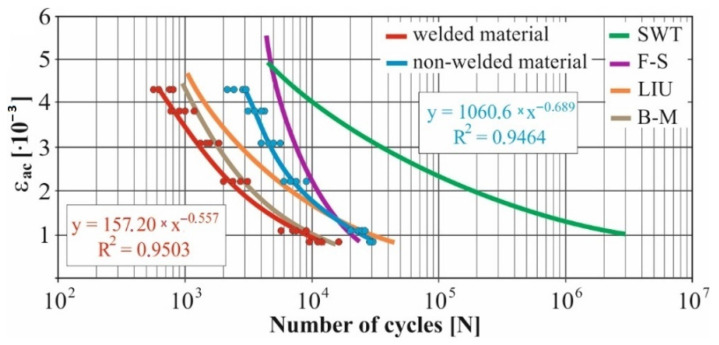
Comparison of *ε_ac_*–*N_f_* curves of selected fatigue criteria and experimentally obtained values, results of low-cycle multiaxial fatigue, constant deformation in torsion *γ_ac_* = 5 × 10^−3^, phase shift of loads *φ* = 90°.

**Figure 12:**
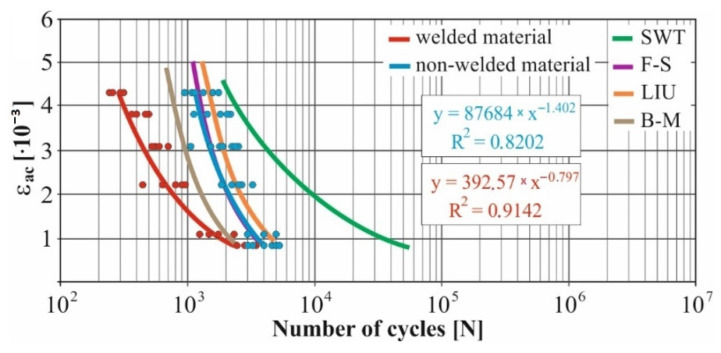
Comparison of *ε_ac_*–*N_f_* curves of selected fatigue criteria and experimentally obtained values, results of low-cycle multiaxial fatigue, constant deformation in torsion *γ_ac_* = 6.9 × 10^−3^, phase shift of loads *φ* = 0°.

**Figure 13:**
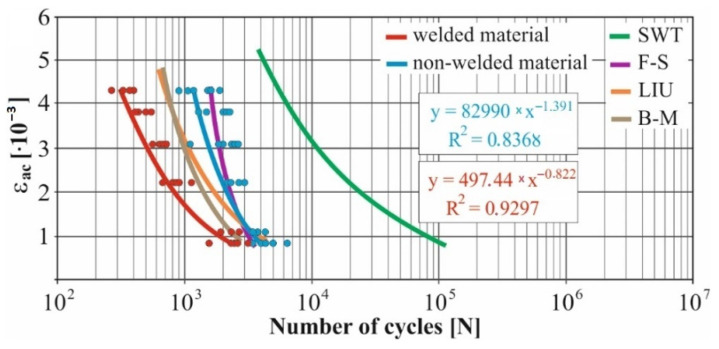
Comparison of *ε_ac_*–*N_f_* curves of selected fatigue criteria and experimentally obtained values, results of low-cycle multiaxial fatigue, constant deformation in torsion *γ_ac_* = 6.9 × 10^−3^, phase shift of loads *φ* = 90°.

**Figure 14:**
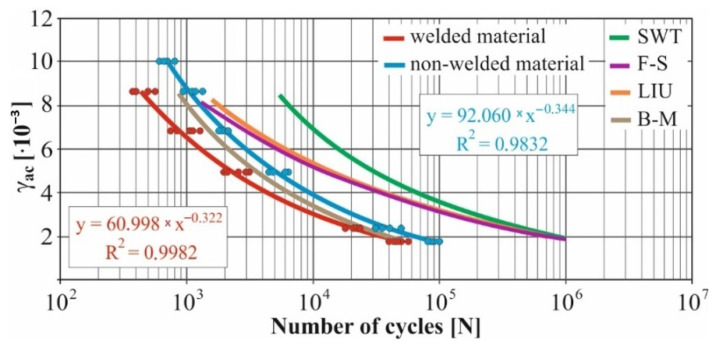
Comparison of *γ_ac_*–*N_f_* curves of selected fatigue criteria and experimentally obtained values, results of low-cycle multiaxial fatigue, constant deformation in bending *ε_ac_* = 2.2 × 10^−3^, phase shift of loads *φ* = 0°.

**Figure 15:**
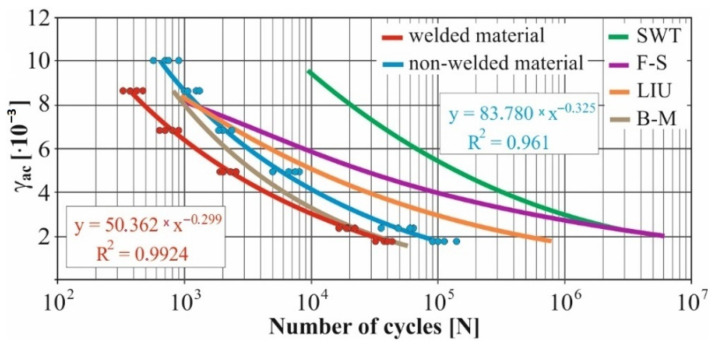
Comparison of *γ_ac_*–*N_f_* curves of selected fatigue criteria and experimentally obtained values, results of low-cycle multiaxial fatigue, constant deformation in bending *ε_ac_* = 2.2 × 10^−3^, phase shift of loads *φ* = 90°.

**Figure 16:**
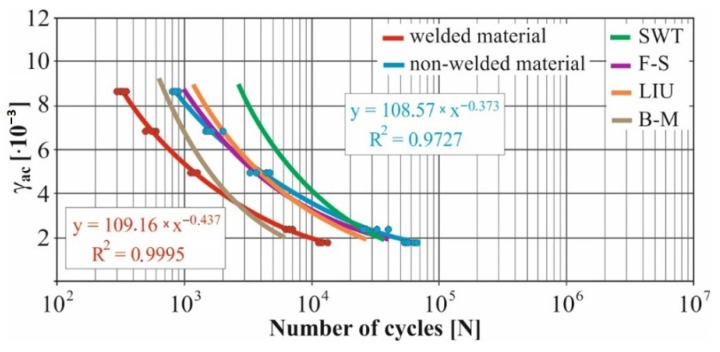
Comparison of *γ_ac_*–*N_f_* curves of selected fatigue criteria and experimentally obtained values, results of low-cycle multiaxial fatigue, constant deformation in bending *ε_ac_* = 3.1 × 10^−3^, phase shift of loads *φ* = 0°.

**Figure 17:**
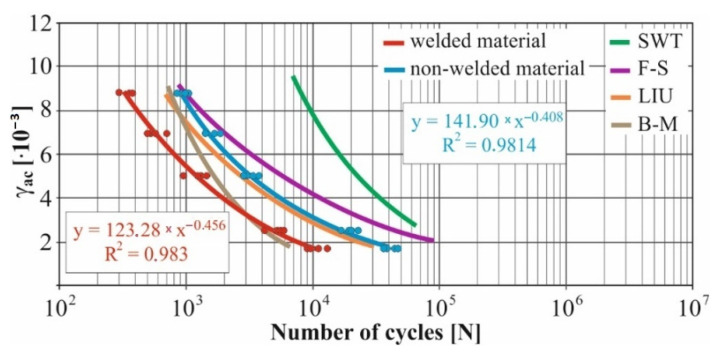
Comparison of *γ_ac_*–*N_f_* curves of selected fatigue criteria and experimentally obtained values, results of low-cycle multiaxial fatigue, constant deformation in bending *ε_ac_* = 3.1 × 10^−3^, phase shift of loads *φ* = 90°.

**Figure 18:**
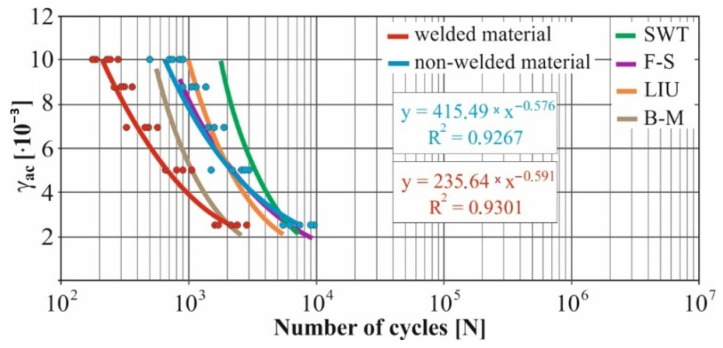
Comparison of *γ_ac_*–*N_f_* curves of selected fatigue criteria and experimentally obtained values, results of low-cycle multiaxial fatigue, constant deformation in bending *ε_ac_* = 3.8 × 10^−3^, phase shift of loads *φ* = 0°.

**Figure 19:**
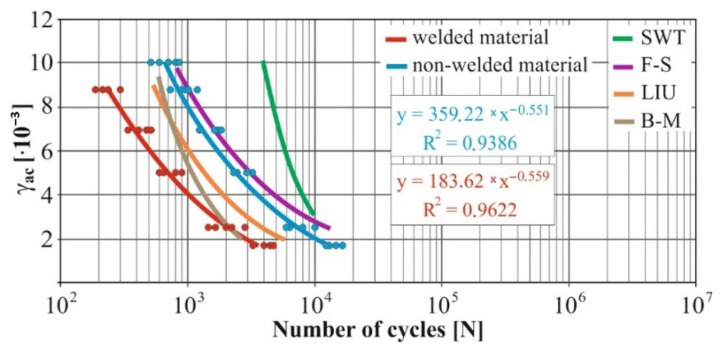
Comparison of *γ_ac_*–*N_f_* curves of selected fatigue criteria and experimentally obtained values, results of low-cycle multiaxial fatigue, constant deformation in bending *ε_ac_* = 3.8 × 10^−3^, phase shift of loads *φ* = 90°.

In real experiments, the number of measurements performed at each level was four. This was true for similar values of the cycle numbers. With a larger scattering of values (observed especially at higher values of deformation amplitudes), the number of measurements reached the value 10. All measured values and values obtained by the Fatigue Calculator program were plotted in graphs (Figure 8, Figure 9, Figure 10, Figure 11, Figure 12, Figure 13, Figure 14, Figure 15, Figure 16, Figure 17, Figure 18 and Figure 19).

## 3. Results

An experimental measurement of the fatigue of the specimens was performed using a Fatigue testing system (Figure 4) as well as a numerical determination using the Fatigue Calculator program. All analyzed data are shown in Figure 8, Figure 9, Figure 10, Figure 11, Figure 12, Figure 13, Figure 14, Figure 15, Figure 16, Figure 17, Figure 18 and Figure 19. 

Experimental results are shown by blue and red curves and results calculated by the Fatigue Calculator program are given by green, violet, orange and brown curves.

The largest differences in the experimentally measured cycles were observed at large deformation amplitudes. The quality of individual welds [15] has a fundamental effect on this fact. With smaller deformation amplitudes, such differences were no longer observed. The measurement of the specimens was performed both for the basic material EN AW 6063 (red line) and for its welds (blue line). This technology will be necessary in the realization of the designed vehicle.

It should be added that the comparison of the results from the program is relevant only with the results from the experiment for the basic material specimens. The same material constants as were used in the calculations of the limit cycle numbers of individual hypotheses in the Fatigue Calculator program are provided for these specimens.

From the graphs above it is possible to observe whether the difference in fatigue between the individual criteria and experiments is more affected by constant deformation in torsion (Figure 8, Figure 9, Figure 10, Figure 11, Figure 12 and Figure 13) or constant deformation in bending (Figure 14, Figure 15, Figure 16, Figure 17, Figure 18 and Figure 19). Damage models, namely the Fatemi–Socie, SWT, Brown–Miller and Liu model, were used in the numerically analyzed low-cycle fatigue. By evaluating the Manson–Coffin curves, it was found that the fatigue curve for the Brown–Miller model under cyclic loading is lower in the whole range of the cycle numbers. From the dependences, it is also possible to observe a relatively good match between the welded material experiment and the Brown–Miller hypothesis especially in the area of low-cycle fatigue.

Furthermore, the results show that there is a difference in the cycle number to the fracture with other monitored dependence as well, i.e., the phase shift between the maxima of the individual loads. For all hypotheses, as well as for the basic material specimen, there is a longer lifetime for the 90° phase [38]. The load on the frame while in operation will be stochastic. Therefore, for future research, the lowest measured values will be taken into account when evaluating the lifetime of the frame material. The difference between the phases practically makes up to approximately 10 to 15% difference in the cycle numbers at the same deformation amplitude. Welded specimens in a few cases withstood more cycles at 0° phase in comparison with the same load at 90° phase. This fact is explained by the uniqueness of each weld in terms of its properties (grain size, heat affected zone area, welding defects) [11].

For all measured deformation amplitudes, the largest difference in the cycle numbers to the fracture occurs between the SWT hypothesis and all other hypotheses and experiments.

## 4. Discussion

To verify the Fatigue testing system calibration, the numerical solutions were based on knowledge of basic material properties. By comparing the results, the outcomes of the experiments are verified. The experimentally obtained data on the welded material provide valuable information for the designers of the vehicle. With simulated frame stresses [10], it is then possible to estimate the fatigue of the welded frame and thus guarantee the safety and reliability of the design during the planned lifespan.

Figure 8, Figure 9, Figure 10, Figure 11, Figure 12, Figure 13, Figure 14, Figure 15, Figure 16, Figure 17, Figure 18 and Figure 19 show that the fatigue curve of the basic material for the Brown–Miller model under cyclic loading is much lower in the whole range of the cycle numbers. This is caused by the range of normal deformation this damage model uses to determine fatigue. It is changed with the ratio of tensile and compressive deformations [39,40]. This fact results in a reduction in fatigue.

The stated criterion is based on the knowledge of deformation parameters (shear and normal deformations). 

These same figures also show that the fatigue curve of the SWT model under cyclic loading is much higher in the whole range of the cycle numbers. Smith, Watson, and Topper created an equation that includes cyclic deformation and maximum stress, and this model was originally developed to correct the mean stress at uniaxial loads. It is now most commonly used in the analysis of proportional and non-proportional loadings. The Smith Watson and Topper parameter is a model based on material caused damage and inherent properties determined from LCF tests [41]. The same coefficient was used for the experimental measurements. This approach can be used to determine fatigue damage caused by components that are subjected to a fully reversed loading. This also applies to the designed vehicle when driving through left and right curves.

The average values of crack initiation and extension times in uniaxial tests were published in the paper [15]. In case of torsion at the smallest measured deformation amplitude of 2.5 × 10^−3^, the crack initiation of the welded and non-welded specimen withstood double the values under the defined experimental conditions. However, the difference in the measured cycle numbers to the fracture caused the crack expansion process. The total time to the fracture at the given lowest level was then 3 times longer in the case of a welded specimen. The same trend was observed in the case of bending. However, during the entire deformation amplitude interval, the fatigue was higher in the basic material. At the highest measured deformation amplitude of 4.3 × 10^−3^, the lifetime of the non-welded sample was on average 3.2 times longer. With decreasing amplitude, this value was reduced to 1.5 times with a deformation of 1.1 × 10^−3^. Comparing the cycle numbers to the fracture of a welded and non-welded specimen made of EN AW6063 material with a phase shift of 0° for low-cycle fatigue (up to 105 cycles), it is clear that the non-welded specimen will have a longer time-to-fatigue. This also supports the results of uniaxial measurements [15], for slightly different material can be found similar result [42]. Based on the findings, it is possible to generalize the conclusions. If possible, it is advantageous not to use welded joints for large deformation amplitudes. Alternatively, when designing and optimizing the vehicle frame, it is necessary to propose one with sufficient rigidity. 

**Figure 20:**
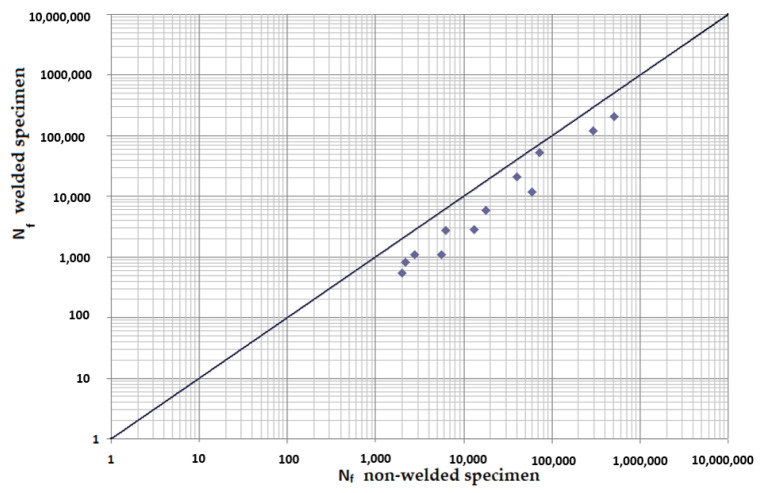
Comparison of experimentally obtained cycle numbers to the fracture of welded and non-welded specimen at phase shift 0°.

**Figure 21:**
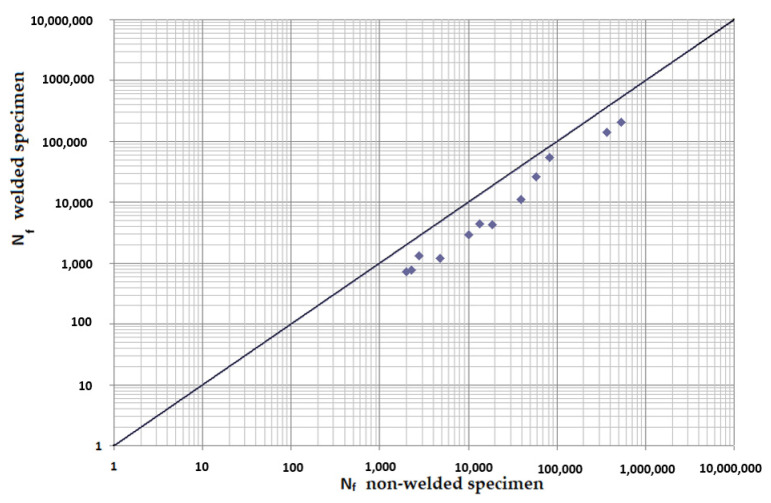
Comparison of experimentally obtained cycle numbers to the fracture of welded and non-welded specimen at phase shift 90°.

Figure 20 and Figure 21 represent a clear comparison of the cycle number to the fracture of the welded and non-welded specimen. They were constructed as dependences on the cycle numbers to the fracture of both specimens at individual phase shifts between loads. Results of measurements up to 500,000 cycles, i.e., where a longer multiaxial lifetime of the non-welded specimen was observed, were used in the construction. Other test conditions, which correspond to Figure 20 and Figure 21, are *γ* (2.5 × 10^−3^; 3.5 × 10^−3^; 3.8 × 10^−3^; 5 × 10^−3^; 6.9 × 10^−3^; 7.9 × 10^−3^; 8.8^−3^; 10 × 10^−3^; 10.7 × 10^−3^), *ε* (1.1 × 10^−3^; 2.2 × 10^−3^; 3.1 × 10^−3^; 3.8 × 10^−3^; 4.3 × 10^−3^) and a symmetrical frequency of 30 Hz. When considering the results from lower deformation amplitudes (results not shown in this paper because they are not essential in terms of safety and critical failures), the measurement proved a higher fatigue of the welded specimen compared to the non-welded one. The situation was similar with a phase shift of 90° between loads (Figure 21). Combinations of higher deformation amplitudes resulted in higher fatigue for the non-welded specimen. On the other hand, for small deformation amplitudes, the fatigue at the combinations of both loads was in favor of the non-welded specimen, which withstands a larger number of cycles compared to the welded specimen. This finding is valuable information in the prediction of multiaxial fatigue at a combination of torsion and bending.

From the number of dependences created for the experimental welded material, which will be thoroughly processed and analyzed, it is already clear that the phase shift will not play such an important role for the welded joints of EN AW6063 aluminum alloy. 

## 5. Conclusions

The presented article deals with experimental evaluation and numerical simulation of fatigue properties of the material EN AW6063. This research is created in order to optimize the design parameters of the frame of an unconventional vehicle. The experimental material will be implemented in the vehicle with the commercial name E3-cycle. The authors investigated the multiaxial fatigue of the basic and welded material in a bending–torsion combination. It was found that:

- The fatigue curve of the basic material for the Brown–Miller model under cyclic loading is lower in the whole range of the cycle numbers. In the case of the given material, the method of its loading and the weld design, the results suggest that the Brown–Miller model can serve as a substitute for this experiment (Figure 8, Figure 9, Figure 10, Figure 11, Figure 12, Figure 13, Figure 14, Figure 15, Figure 16, Figure 17, Figure 18 and Figure 19);
- In the basic material, a higher fatigue was observed at a phase shift of 90°. The difference between the phases practically makes up to approximately 10 to 15% difference in the cycle numbers at the same deformation amplitude (Figure 9, Figure 11, Figure 13, Figure 15, Figure 17 and Figure 19);
- In the welded material this phenomenon was not confirmed, i.e., the stochastic properties of welded joints have the greatest effect on the fatigue of welds (Figure 9, Figure 11, Figure 13, Figure 15, Figure 17 and Figure 19);
- Fatigue of specimens responds more sensitively to a small change in the deformation amplitude in comparison with torsion;
- Software developers in this specific field can use obtained data to expand the material database;
- In the area of low-cycle multiaxial fatigue (up to 5 × 10^5^ cycles), the fatigue of the basic material is higher in comparison with the weld (Figure 20 and Figure 21).

Conclusions will be decisive in the frame design optimization of the presented vehicle, i.e., used in a short time in a real technical solution. They will serve as input parameters in the static and dynamic analysis of the designed vehicle frame.

## Data Availability

Not applicable.

## Nomenclature

TIG	Non-melting tungsten electrode welding in the inert gas atmosphere
1F2R	Delta layout for three-track vehicle
*M_o_*	Bending moment
*M_oF_*	Bending moment from the engine of experimental mechanism
*M_oB_*	Reaction bending moment in the experimental specimen holder
*D*	Diameter of specimen out of the tensile concentrator area
*d_o_*	The smallest diameter of the specimen in the area of tensile concentrator
*d_y_*	General diameter (variable in length) of the specimen in the tensile concentrator area
*x*	Horizontal axis of the coordinate system
*y*	Vertical axis of the coordinate system
*s*	Distance of the curvature center of the circle needed to form the specimen tensile concentrator from the horizontal axis (x)
*l*	Specific length of tensile concentrator for the specimen
*R*	The radius of circle curvature needed to form the tensile concentrator of the specimen
*R* ^2^	Coefficient of regression model determination
*R_A_*	Supportive reaction in connection point A
*R_B_*	Supportive reaction in connection point B
U	Tensile energy (internal energy)
*Ε*	Modulus of elasticity in tension
*N_f_*	Number of cycles to fracture
*γ_ac_*	Amplitude of total deformation in shear
*W_o_*	Bending cross-sectional modulus
*J_z_*	Moment of inertia
SWT	Fatigue criterion according to Smith, Watson and Topper
F-S	Fatigue criterion according to Fatemi–Socie
LIU	Fatigue criterion according to Liu
B-M	Fatigue criterion according to Brown–Miller
*ε_ac_*	Amplitude of total bending deformation
*φ*	Phase shift of load
*σ_xx_*	Bending stress (normal)
*τ_xy_*	Torsional stress (tangential)
